# Integrating ‘NeedCraft’ and ‘NeedNurture’: a dual-level realist-informed intervention protocol to enhance basic psychological needs at work

**DOI:** 10.3389/fpsyg.2026.1739446

**Published:** 2026-08-05

**Authors:** Ivan Putter, Leoni van der Vaart, Jacqueline Bosman

**Affiliations:** 1 Optentia Research Unit, North-West University, Vanderbijlpark, South Africa; 2 Department of Psychology, Norwegian University of Science and Technology, Trondheim, Norway

**Keywords:** autonomy, basic psychological need satisfaction, competence, positive psychology, relatedness, self-determination theory, realist evaluation

## Abstract

**Purpose:**

From a self-determination theory (SDT) perspective, need crafting holds that the basic psychological needs for autonomy, competence, and relatedness can be proactively shaped through intentional changes in behavior and cognition. This protocol outlines the development of an SDT-based intervention targeting both employees [‘NeedCraft’ (NC)] and their managers [‘NeedNurture’ (NN)] to enhance need-based experiences in a South African mining organization.

**Methods:**

The protocol proposes a quasi-experimental mixed-methods design with a partially randomized delayed-start, assigning departments to an immediate (Group 1: NC + NN), delayed (Group 3: NC + NN), or non-randomized (Group 2: NC only) condition. Effect evaluation will test whether the intervention improves need crafting, perceived leader support, and need satisfaction over time, using linear mixed-effects models with proximal and distal outcomes capturing short- and longer-term improvements. Complementing this, a realist-informed process evaluation is proposed to examine context-mechanism-outcome configurations to explain how, why, and under what conditions change occurs.

**Anticipated results:**

The intervention is expected to produce short-term improvements in employees’ need crafting behaviors and in perceived need-supportive leader behaviors (proximal outcomes), followed by longer-term improvements in basic psychological need satisfaction (distal outcome), with the dual-level condition (NC + NN) expected to outperform the employee-only condition.

**Conclusion:**

The protocol positions NC and NN as positive psychological interventions and aims to advance theoretical, methodological, and practical understanding of how to design, implement, and sustain need-based interventions in complex organizational settings.

## 1 Introduction

Work engagement – a positive, fulfilling work-related state of mind characterized by vigor, dedication, and absorption – reflects the extent to which employees invest their full selves in their work roles (Kahn, 1990; Schaufeli et al., 2002). Nevertheless, according to Gallup’s (2025) State of the Global Workplace report, only 21% of employees worldwide were engaged in 2024. Gallup estimates that the decline in engagement that year cost the world economy US$438 billion in lost productivity, whereas fully engaged workplaces could add US$9.6 trillion (≈9% of global gross domestic product). The situation is particularly relevant in sub-Saharan Africa, where only 19% of employees are engaged. These figures underscore the need for contextually appropriate interventions to strengthen work engagement. Accordingly, the primary objective of this protocol is to describe the design, implementation, and evaluation plan for a dual-level intervention, grounded in self-determination theory (SDT), in a South African mining organization. The intervention simultaneously teaches employees to proactively craft satisfaction of their basic psychological needs (‘NeedCraft’) and trains their managers to support those needs (‘NeedNurture’).

The proposed intervention addresses the substantial individual and organizational consequences of employee engagement. Lower engagement is linked to declines in performance (Mokgata et al., 2023; Rothmann and Rothmann, 2010; Ryan and Deci, 2017) and higher turnover intentions (Putter et al., 2024). This pattern aligns with Gallup’s (2025) finding that 50% of employees intend to leave their organizations, rising to 72% in sub-Saharan regions. Conversely, when engagement increases, research shows that employees are more likely to exert effort and less likely to leave, which, in turn, is associated with organizational success and profitability (Bailey et al., 2017; Mackay et al., 2017).

In addition to widespread engagement challenges, the 2023 Well-Being at Work Survey, conducted by Deloitte and Workplace Intelligence and reported by Fisher et al. (2023), identified a manager–employee perception gap. Although employees indicated that several aspects of their well-being had stagnated or worsened, more than three-quarters of managers believed that their employees’ well-being had improved. This discrepancy reveals a disconnect between employees and management, which can weaken engagement unless organizations empower managers to actively support their subordinates’ well-being, particularly because perceived organizational support is positively associated with employees’ emotional and physical well-being (Biedma-Ferrer et al., 2024).

Drawing on self-determination theory (SDT) (Deci and Ryan, 1985; Ryan and Deci, 2017), social environments – particularly managerial support – play a pivotal role in fulfilling employees’ three basic psychological needs: autonomy, competence, and relatedness (Ryan and Deci, 2017; Slemp et al., 2021). Autonomy involves experiencing volition and the power to make choices. Competence entails experiencing mastery and effectiveness. Relatedness involves feeling significant to others and having a sense of connection with them (Vansteenkiste et al., 2020). When these needs are met, employees are more likely to feel engaged, perform effectively, and remain with their organization (Ryan and Deci, 2017; Slemp et al., 2021; Van den Broeck et al., 2016). Satisfied needs may also foster employees’ capacity to thrive at work – a malleable, state-like experience characterized by vitality and learning and responsive to individual and supportive social conditions (Arici Ozcan et al., 2023; Spreitzer et al., 2005). From a positive psychology stance, the present protocol takes a capability-building approach that aims to cultivate conditions in which optimal need-based experiences can emerge and endure (see Ryan and Deci, 2000; Sheldon and Ryan, 2011; Vansteenkiste et al., 2020). Cross-cultural evidence indicates that these three needs function as universal psychological nutriments, predicting well-being and adaptive functioning across diverse settings (Duan et al., 2022), supporting SDT-framed intervention in non-WEIRD (Western, educated, industrialized and rich) organizational settings (Sheldon, 2012; Duan et al., 2022; Van der Vaart et al., 2026), including South African workplaces.

Moreover, employees are motivated to satisfy their basic psychological needs and improve their circumstances by proactively seeking experiences aligned with these needs (Kotze et al., 2024; Laporte et al., 2021; Ryan and Deci, 2017). This proactive behavior is known as need crafting (Kotze et al., 2024; Laporte et al., 2021) and aligns with the notion that the basic psychological needs are directional (Vansteenkiste et al., 2020). Work-related need crafting involves deliberate cognitive and behavioral changes to job content or the context of work in order to satisfy one’s basic psychological needs at work (Kotze et al., 2024; Olafsen et al., 2025). It differs from job crafting (Tims and Bakker, 2010; Wrzesniewski and Dutton, 2001), which primarily focuses on altering tasks/roles or job demands/resources to find meaning and fit within one’s job. Unlike job crafting, work-related need crafting specifically aims to satisfy basic psychological needs (Putter et al., 2024), treating changes in job characteristics as distal rather than proximal outcomes (Olafsen et al., 2025). This needs-first lens also captures practices often missed in job crafting measures (e.g., non-instrumental relatedness and value-based autonomy behaviors) (Olafsen et al., 2025). Consequently, work-related need crafting interventions are health-promotion efforts, rather than illness-prevention programs.

Previous need crafting studies have reported positive well-being outcomes (Behzadnia and FatahModares, 2020, 2023; Laporte et al., 2024; van den Bogaard et al., 2024; Weinstein et al., 2016). However, these intervention studies can be supplemented in several ways. Firstly, most studies (Behzadnia and FatahModares, 2020; Laporte et al., 2024; van den Bogaard et al., 2024; Weinstein et al., 2016) focused on non-work contexts. While recent research indicates that need crafting is relevant in work settings, its impact may vary due to contextual factors (Mokgata et al., 2023; Putter et al., 2024). Secondly, some studies (Behzadnia and FatahModares, 2020; Weinstein et al., 2016) were conducted under highly stressful conditions, such as refugees resettling in a new country or people isolating during the COVID-19 pandemic. It remains unclear whether individuals will continue to engage in need crafting during everyday difficulties and whether they will experience equally positive outcomes.

Thirdly, some studies reported mixed (Behzadnia and FatahModares, 2020) or minimally positive (Weinstein et al., 2016) outcomes, making it difficult to draw definitive conclusions regarding the effectiveness of need crafting interventions. In the fourth place, the studies focused primarily on the individual level, which may have contributed to the minimal changes reported. Interventions that focus solely on the individual often fail to consider the active involvement of key stakeholders at multiple levels beyond the individual (De Lange et al., 2024). In the fifth place, to our knowledge, no published, peer-reviewed standardization protocols were available for these interventions. Although tailored interventions may enhance relevance, they complicate the identification of factors that contribute to intervention effects (Abildgaard et al., 2016). To this extent, Slemp et al. (2021) recommend standardized intervention protocols or conceptual guidelines to facilitate treatment fidelity. Lastly, those studies that included process measurement focused solely on treatment fidelity, usefulness, and intervention engagement at the individual level (Behzadnia and FatahModares, 2020, 2023; Laporte et al., 2024; van den Bogaard et al., 2024; Weinstein et al., 2016). This is problematic because it neglects critical multilevel dynamics and broader contextual factors that influence intervention outcomes.

To address these limitations, we propose an integrated intervention combining ‘NeedCraft’ (NC) and ‘NeedNurture’ (NN) to increase opportunities for autonomy, competence, and relatedness. This dual approach combines a proactive bottom-up strategy through individual employees’ need crafting (NC) with a top-down strategy through supervisors’ need-supportive behaviors (NN). This paper presents the study protocol to guide the subsequent design, implementation, and evaluation of the multilevel intervention. Specifically, the protocol employs a dual evaluation strategy: (1) process evaluation to examine stakeholders’ perceptions and actions before, during, and immediately after implementation (Nytrø et al., 2000) and (2) outcome evaluation to capture the effect of the intervention on the target population (Slemp et al., 2021). By examining both process and content mechanisms, this protocol will explore how the intervention may attain its desired outcomes and specify for whom and why these outcomes may vary (Nielsen and Miraglia, 2016). In addition to examining process-related mechanisms, the outcome evaluation specifically targets proximal outcomes (i.e., need crafting and need support), which are considered direct results of these interventions, as well as distal outcomes (i.e., need satisfaction).

## 2 Methods

### 2.1 Study design

The current intervention protocol recommends a quasi-experimental mixed-methods design using a partially randomized delayed-start approach with three experimental groups. Departments (clusters) will be randomized to Group 1 (NC + NN) or Group 3 (delayed NC + NN); departments whose managers do not volunteer will form Group 2 (NC only, non-randomized). Group 2 will demonstrate the superiority of a multilevel intervention (i.e., its cumulative effects), whereas Group 3 will serve as a control group until its members have received the intervention.

### 2.2 Setting and participants

A South African earth-drilling and rock-boring organization with 612 employees (Identity hidden to protect the organization, personal communication, July 28, 2022) was purposively selected for the current study because it offers a non-WEIRD, high-demand context in which autonomy, competence, and relatedness are structurally constrained by safety protocols, hierarchical supervision, and shift work. Although it is a global organization, only the South African head office was included in this study. We limited the study to the South African head office to reduce cross-site heterogeneity in labor law, language, HR policies, and safety procedures; to minimize contamination across clusters; and to ensure feasibility for training delivery, process monitoring, and data protection in line with the Protection of Personal Information Act (POPIA). Focusing on one site increases internal validity and protocol fidelity while preserving ecological validity in a real-world mining services environment. Prior formative work (Putter et al., 2024) with this site established access, organizational readiness, and outcome relevance, further justifying site selection.

The current study will rely on criterion (purposive) sampling, in which participants from selected organizational departments and/or their direct line managers will be included based on specific, predetermined inclusion criteria relevant to the study. For South Africans to be considered employed, they should be “between the ages of 15 and 64” (Statistics South Africa [Stats SA], 2022, p. 2). However, for ethical reasons, participants should be at least 18 and hold at least a Grade 12 certificate to optimally grasp the content of the need-crafting intervention training. The researchers also considered it appropriate that participants had been employed in their position for at least 1 month. Meta-analytic results show a negative association between tenure and job crafting (Rudolph et al., 2017), which may be ascribed to the fixed mental schemas of employees with longer tenure and the resultant impact on behavior (Zacher and Kooij, 2017). At the same time, experience may put them in a better position to craft, as they will know “what” and “how” to craft (Rudolph et al., 2017). Finally, only white-collar employees will be included in the intervention because of the inherent safety risks associated with crafting, for example, among machine operators in the mining sector. The daily operational duties of machine operators generally allow for limited crafting in light of mining safety protocols.

The individual, group, leader, and organizational (IGLO) model proposed by Nielsen et al. (2017) suggests that interventions target the individual, group, leader, and organizational levels. The IGLO model states that an individual-level intervention will have a negligible impact if it does not also address another organizational level (e.g., leaders). Nielsen et al. (2023) suggest that individual-level interventions should focus on enhancing and upholding an employee’s well-being through activity engagement, building personal resources, developing resilience, and ensuring positive coping mechanisms. Given managers’ ability to support their subordinates’ needs (see Slemp et al., 2021 for an overview), the individual-level intervention will be complemented by a leader-level intervention. At the time of NC, the NN intervention (see Tables 1, 2) will offer parallel training to managers, equipping them to support their employees’ basic psychological needs.

**TABLE 1:** Overview of ‘NeedCraft.’

Steps	Aspects of the intervention that reflect Kolb’s experiential learning theory	Example activities (targeted needs)
Step 1: concrete experience (feeling)	∙ Participants identify and reflect on their current need satisfaction experiences at work. ∙ They engage in exercises to recognize moments when their psychological needs are met or frustrated.	∙ Participants recall recent workdays and identify moments in which they felt effective, connected to others, or free to choose, e.g., re-doing a task that was a slight but manageable challenge (competence).
Step 2: reflective observation (watching)	∙ There is guided reflection on these experiences, analyzing what contributes to need satisfaction or frustration. ∙ Participants discuss how current work practices have an impact on their psychological needs.	∙ Participants share a meaningful work experience with a colleague and reflect on how giving and receiving appreciation affected them (competence, relatedness).
Step 3: abstract conceptualization (thinking)	∙ Participants are introduced to need crafting concepts and strategies. ∙ Participants learn theoretical frameworks (SDT) for understanding psychological needs and how to craft experiences to meet them.	∙ Participants review a 10-days menu of need crafting activity ideas, each linked to the needs it targets, e.g., teaching a colleague a new skill (competence, relatedness); making a meaningful choice about how the day is structured (autonomy); finding a new or creative way to do a routine task (competence); engaging in positive, appreciative dialogue with a colleague (relatedness).
Step 4: active experimentation (doing)	∙ Participants develop personalized need crafting plans (using the SMART framework to ensure strategies that are specific, measurable, achievable, realistic, and time bound). ∙ Participants brainstorm on enablers and barriers to applying need crafting strategies. They discuss how to optimize enabling factors and mitigate barriers (i.e., inoculation against setbacks). ∙ Participants implement these strategies in their daily work over a set period (i.e., 2 weeks). ∙ After the implementation period, participants reconvene to share experiences, reflecting on the effectiveness of their need crafting efforts and how they can sustain this in the organization.	∙ Participants translate selected activities into SMART goals in their personal need crafting plan, e.g., “walk and talk with a colleague during lunch twice this week” (relatedness); “discuss changes to my schedule with my manager” (autonomy); “offer more input during meetings” (competence, autonomy); “start meetings by asking how everyone is doing” (relatedness).

Adapted from Demerouti et al. (2021).

**TABLE 2:** Overview of ‘NeedNurture.’

Steps	Aspects of support that reflect Kolb’s experiential learning theory	Example activities (targeted needs)
Step 1: concrete experience (feeling)	∙ Participants identify and reflect on their current need-supportive “communication” at work. ∙ They engage in exercises to recognize moments when their actions positively or negatively affect employees’ needs.	∙ Managers recall recent interactions in which their words or actions visibly affected an employee’s motivation, e.g., how they responded when an employee shared an idea, concern, or mistake.
Step 2: reflective observation (watching)	∙ There is guided reflection on these behaviors, analyzing what contributes to need satisfaction or frustration. ∙ Participants discuss how their management practices influence psychological needs.	∙ Managers reflect on critical incidents from their own practice, e.g., keeping reactions to employees’ contributions affirming and uplifting rather than belittling (relatedness and competence support).
Step 3: abstract conceptualization (thinking)	∙ Managers are introduced to SDT to explain the role of autonomy, competence, and relatedness in fostering employee motivation. ∙ Managers are taught specific strategies to become more need supportive (e.g., offering meaningful choices to employees, providing constructive feedback, and fostering a sense of belonging in the team).	∙ Managers work through strategy examples linked to each need: giving employees input and say in schedules, deadlines, and meeting arrangements (autonomy support); offering coaching or mentoring and competence-affirming feedback (competence support); engaging in appreciative dialogue and regular one-on-one check-ins (relatedness support).
Step 4: active experimentation (doing)	∙ Managers create personalized strategies (using the SMART framework to ensure strategies that are specific, measurable, achievable, realistic, and time bound). ∙ Participants brainstorm on enablers and barriers to applying need-support strategies. They discuss how to optimize enabling factors and mitigate barriers (i.e., inoculation against setbacks). ∙ Managers implement these strategies in their daily work over a set period (e.g., 2 weeks). ∙ After the implementation period, participants reconvene to share experiences, reflecting on the effectiveness of their need-supportive efforts and how they can sustain this in the organization.	∙ Managers translate selected strategies into SMART goals in their personalized need-support plan, e.g., “involve employees in the setting of deadlines and targets” (autonomy support); “help an employee brainstorm a new way to approach a problem” (competence support); “start meetings by asking how employees are doing” (relatedness support).

Adapted from Demerouti et al. (2021).

### 2.3 Preparing participants for the study

The researcher, in collaboration with designated human resources (HR) gatekeepers, will first address departmental heads, followed by similar in-person information sessions for groups of departmental employees. These sessions will provide detailed information about the study’s purpose, requirements, confidentiality measures, and the voluntary nature of participation. Interested individuals will then sign up for participation by providing their preferred contact details (i.e., email address), allowing the researcher to distribute questionnaires via Google Forms at various points throughout the intervention and to facilitate future study-related communication. A POPIA-compliant process will be followed to obtain consent to process these contact details, ensuring the lawful processing of personal information (i.e., “accountability, processing limitation, purpose specification, further processing limitation, information quality, openness, security safeguards, data subject interpretation”) (Presidency of the Republic of South Africa, 2013, p. 18). In accordance with POPIA and the Health Professions Council of South Africa [HPCSA], 2016) research-data guidelines, all de-identified data, linked only to participants’ unique identification (ID) codes, will be stored on a password-protected, NWU-approved drive for a minimum of 5 years after study completion, after which it will be securely deleted; any data shared with collaborators will remain de-identified.

The consent form will explicitly outline the lawful use of participants’ personal details (such as email addresses) solely for distributing online questionnaires via Google Forms at designated intervals, sending reminders, arranging follow-up meetings, and distributing process and outcome evaluation assessments. All participant information will remain confidential, stored on a password-protected device and accessible only to the research team. To ensure confidentiality, participants will be required to generate unique identification codes, which will be used exclusively to match their questionnaire responses across measurement intervals. No identifying personal details will be provided to the host organization.

Employees will also be asked to identify the department in which they work. The researcher will then randomly assign a number to each department. Similarly, managers will be asked which department they manage, allowing the researcher to allocate the corresponding number to each manager (e.g., accounting department = 1, training department = 2, etc.). Consequently, no names will be required to match departments with managers, and all participants will be reassured of anonymity and confidentiality, since no identifying information will be collected. This will enable employee participants to engage in the need-crafting intervention while also allowing the respective managers to engage in the need-support intervention simultaneously.

**FIGURE 1:**
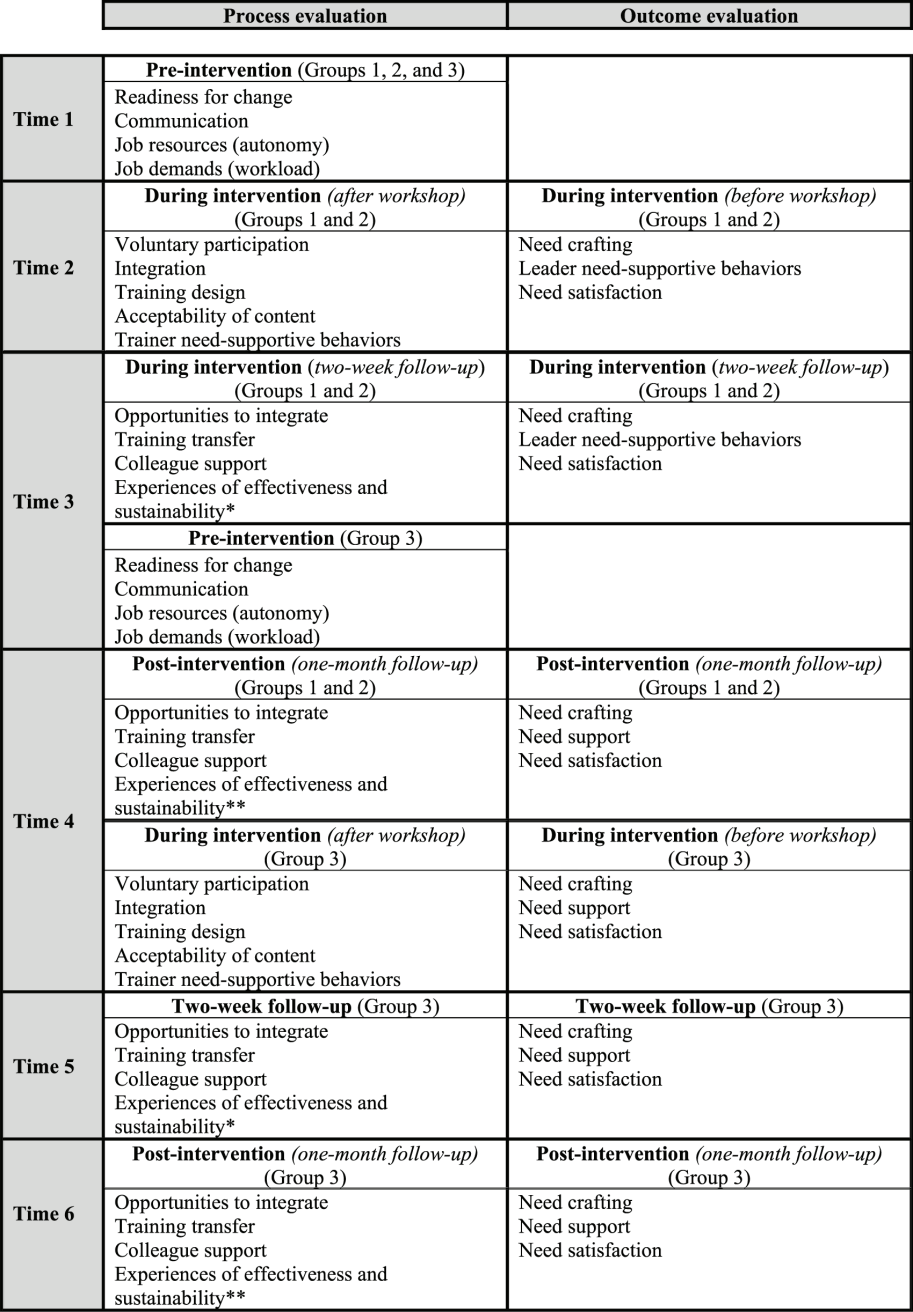
The integrated process evaluation framework (IPEF). Adapted from Nielsen et al. (2023, p. 17) and De Angelis et al. (2020, p. 13). *Focus group discussions. **Open-ended surveys.

During the in-person training sessions, all participants will be asked to complete hard copies of the provided questionnaires using their unique IDs. Subsequent follow-up measures for Groups 1 and 2 (T4) and Group 3 (T6) (Figure 1) will be distributed electronically by the researcher.

Employees feel more empowered when they see their managers engage in mutual learning. Such managerial participation may also contribute to stronger relationships among employees and between employees and their managers. Based on this, the researcher will encourage and motivate managers to include and involve their departmental employees in the intervention study, thereby strengthening working relationships and promoting personal empowerment. The researcher will hold departmental meetings with managers and employees to ensure that the intervention study is communicated equally to all prospective participants. Managers will also attend separate meetings and be informed of the potential advantages and challenges associated with intervention studies within organizations to help empower them to motivate and support their employees in participating in the study.

When recruiting participants for the intervention study, encouraging engagement can be challenging, particularly when participants come from diverse cultural and socio-economic backgrounds (Olem et al., 2009), such as those in South Africa (Olem et al., 2009). Non-adherence to intervention activities can undermine the study’s internal validity, skew results, and make interpretation difficult or inaccurate. Such outcomes can ultimately increase the likelihood of the intervention study being regarded as ineffective.

Recognizing the challenges of sustaining participant engagement in intervention studies (Olem et al., 2009) – especially in diverse contexts such as South Africa – the researcher will proactively encourage participation. Specifically, the researcher will emphasize that participation is voluntary and confidential, and clearly distinct from organizational mandates. Potential doubts or hesitations participants may experience will be openly addressed and normalized. During training and orientation, participants will be invited to identify potential barriers to their engagement, enabling the researcher to collaboratively problem-solve these issues (see Tables 1, 2 for details). Participants will also receive the researcher’s direct contact information, ensuring accessible support and assistance. The researcher will emphasize respect and appreciation for each participant’s contribution and clearly communicate how individual involvement will benefit overall study outcomes and personal development. Regular contact and reminders will reinforce participants’ sense of inclusion and engagement throughout the study, enhancing ongoing commitment (Buchanan and Hvizdak, 2009).

The researcher will also do check-ins with participants to maintain awareness of the study. Research has shown that participants are more likely to engage with research if they are reminded (Buchanan and Hvizdak, 2009). All personal information will only be used for research purposes, accessible only to the research team, and will be destroyed after feedback. The research study has ethics clearance from the North-West University’s Economics and Management Sciences Research Ethics Committee (NWU-01874-22-A4).

### 2.4 Sample size and power

It is important to determine the optimal sample size to enable effective statistical analysis. Accordingly, an *a priori* power analysis using G*Power indicated that at least 26 participants per group would be required for a large effect (Cohen’s *d* = 0.80) and 51 for a medium effect (Cohen’s *d* = 0.50). These estimates were based on an alpha level of 0.05 and 80% power (Faul et al., 2007).

### 2.5 Allocation to intervention

All departments that sign up will be treated as separate clusters, followed by randomization (e.g., using a random number generator) to assign each department to either Group 1 (immediate intervention) or Group 3 (delayed intervention). Participants without participating managers will automatically be assigned to Group 2.

### 2.6 Intervention content and delivery

#### 2.6.1 ‘NeedCraft’ and ‘NeedNurture’

The current study involves two interventions designed to enhance need satisfaction in the workplace. NC is designed to teach employees how to craft their work experiences to better meet their psychological needs. In parallel, NN targets managers, aiming to upskill them to support their subordinates’ basic psychological needs. This standardized protocol will guide the design, effective implementation, and evaluation of the interventions and will also aid stakeholders and managers in future action orientation through more effective decision-making about employee well-being, policy design, etc., (De Angelis et al., 2020).

Both interventions are grounded in Kolb’s learning theory (Kolb, 1984), which holds that effective learning occurs through four stages (see Tables 1, 2). First, learners engage in concrete experiences, followed by reflection on these experiences in the second stage. In the third stage, learners develop abstract ideas about new behaviors and acknowledge their potential benefits. Finally, they actively test these new behaviors, creating new experiences (Demerouti et al., 2021; Kolb et al., 2001; Nielsen and Randall, 2013). NC and NN incorporate these four stages into their learning processes to ensure comprehensive skill development and behavioral change.

The training will be delivered in person, as research shows that face-to-face training tends to yield higher participant satisfaction compared to online formats (Laporte et al., 2024). The program structure consists of an initial 2.5-h training session (see Steps 1–3 in Tables 1, 2), followed by 2 weeks to implement newly acquired skills and behaviors in the workplace. After this implementation phase, a 2-h reflection meeting will be held (see Step 4 in Tables 1, 2) to discuss experiences, reinforce learning, and adjust strategies as needed.

In the NC intervention (see Table 1), employees are guided through complementary activities that help them become more aware of opportunities and resources to satisfy their psychological needs (Laporte et al., 2021). Such activities can include individual employees negotiating opportunities for more independent decision-making with their supervisor (autonomy crafting), reflecting on successes (competence crafting), or initiating a social activity (relatedness crafting) (Kotze et al., 2024). During the 2.5-h intervention training session, participants will be expected to reflect on their own jobs through a job analysis. Such a job analysis speaks to employees’ involvement, proactivity, and improved performance in their jobs (Rudolph et al., 2017) and will help them identify and relate to the need crafting training and its applicability to their daily tasks more easily. Through this, they will be better equipped to identify opportunities to initiate and apply the newly acquired need crafting capabilities in their everyday jobs.

As part of the need crafting training, participants will be introduced to the concept of need crafting, as well as autonomy, competence, and relatedness crafting, and SMART (specific, measurable, achievable, realistic, and time-bound) goal setting. Toward the end of the 2.5-h intervention training session, participants will be required to apply this newly acquired knowledge to compile a personal need crafting plan (employees). Examples of need-specific activities for each stage of the NC training, drawn from the participant workbook, are provided in Table 1.

At the same time, the NN intervention (see Table 2) offers parallel training for managers, equipping them to support their employees’ basic psychological needs. When managers are more need-supportive toward employees, through behaviors such as encouragement, appreciation, and showing understanding, they promote employees’ need-based experiences. However, managerial behaviors such as criticism, rejection, excessive pressure, or simple neglect and ignoring can have the opposite effect on employees’ need-based experiences (Huyghebaert-Zouaghi et al., 2023). Indeed, employees who believe that speaking up may embarrass their supervisors or have negative career consequences are more likely to engage in defensive silence, while perceived power distance is independently associated with such silence (Şahin et al., 2021). These implicit beliefs may persist even when managers appear to encourage employee voice (Detert and Edmondson, 2011). Deliberate managerial training may therefore help managers communicate consistently that employee initiative, including proactive need crafting, is valued and will not be penalized.

Examples of the need-supportive behaviors practiced during each stage of the NN training are provided in Table 2.

This dual-level approach aligns with the IGLO model, which emphasizes targeting multiple organizational levels to maximize intervention impact. Focusing solely on individuals may limit overall impact if other levels, such as leadership or organizational policies, are not addressed simultaneously (Nielsen et al., 2023).

Applying the IGLO model, NC targets the individual level by helping employees enhance their need-based experiences through activities that build personal resources and positive coping mechanisms. Research highlights the positive impact of need crafting interventions at the individual level (Laporte et al., 2024; van den Bogaard et al., 2024). At the leader level, NN focuses on training direct managers to support subordinates’ basic psychological needs. Research also highlights the positive impact of need-support from others. For example, employees who reported their managers as more need-supportive felt better (Huyghebaert-Zouaghi et al., 2023) and more motivated (Van der Vaart et al., 2025). Colleagues constitute an additional source of support: collegial solidarity has been associated with job performance partly through self-efficacy, work engagement, and thriving at work, although a smaller direct association remains (Karataş and Çankır, 2023). This finding is consistent with the pathway targeted in the present protocol, whereby supportive social conditions may foster valued outcomes through employees’ psychological experiences.

Both interventions will rely on goal-setting to strengthen participants’ commitment to crafting their needs. Participants will be required to develop SMART goals as part of their need crafting plans (see Demerouti et al., 2021 for a job crafting intervention). Specific, sufficiently challenging goals can direct attention and effort toward goal-relevant activities and promote persistence, particularly when participants are committed to those goals and receive feedback on their progress (Locke and Latham, 2002). Where appropriate, need-crafting plans will incorporate both personal and organizational objectives, as job crafting interventions that combined these objectives showed moderate effectiveness in improving work engagement (Oprea et al., 2019; Roczniewska et al., 2022).

In conclusion, this intervention protocol focuses on both individuals (i.e., employees) and leaders (i.e., managers), recognizing that employees can occupy different roles (e.g., employee, manager, or colleague). At the individual level, employees (in their role as subordinates) will create a need crafting plan, while leaders (in their role as managers) will develop a need-support plan. This alignment, alongside improved communication and shared standards, is expected to enhance decision-making and contribute to both organizational and individual success (Randall et al., 2009).

The successful transfer and sustainability of training are dependent not only on support outside of the training session, but also on the physical environment in which intervention training takes place (Grossman and Salas, 2011). The trainer’s education and experience contribute significantly to the success of knowledge transfer during intervention training (Nielsen et al., 2023; Vasquez et al., 2026). As a result, the trainer for the interventions will be a qualified and registered organizational psychologist who will provide optimal training support as well as establish conducive and private training environments at the participants’ place of employment. Building on Van der Vaart and Van den Broeck’s (2019) recommendations, the trainer is expected to model need-supportive behaviors throughout the intervention. By experiencing freedom of choice in developing need crafting strategies and understanding the rationale behind the intervention (i.e., autonomy support), participants are more likely to experience autonomy satisfaction. To foster competence support, the trainer will normalize potential challenges that may arise as participants engage in need crafting activities (i.e., inoculation against setbacks) and help them feel competent to address these. Finally, the trainer should actively demonstrate warmth, validation, and genuine support (i.e., relatedness support) so that both managers and employees experience a genuine sense of belonging and acceptance (Slemp et al., 2021).

### 2.7 Data collection and timeline

**FIGURE 2:**
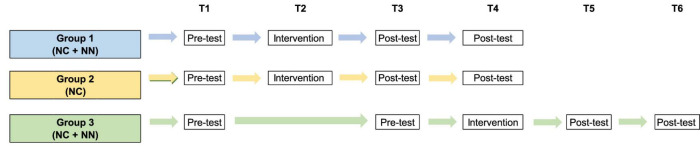
Study design. NC, ‘NeedCraft’; NN, ‘NeedNurture.’

As depicted in Figure 2, all three groups will undergo the same pre-testing simultaneously (before any intervention training commences), and it will comprise the following stages: allocate departments to three groups; perform the pre-test data collection on all three groups at the same time and under the same conditions (T1); provide the first two experimental groups with the intervention supplemented by process and outcome measurements (T2); and provide the delayed experimental group with another baseline measurement of the same pre-test assessment battery to account for any possible changes that may have occurred in the time after they were part of the initial pre-test data collection (T3), as well as commence with post-testing on the first two groups (T3). Thereafter, the third group will receive the intervention (T4), followed by post-testing on all three groups 2 weeks after the training and 1 month after they have completed their intervention training to determine the potential impact on the dependent variable (need satisfaction) within each group as well as measure the possible differences between the groups due to delayed intervention delivery. Each group will undergo two separate post-tests, with all post-testing occurring at four different time points (Demerouti et al., 2021; Laporte et al., 2021).

### 2.8 Effect evaluation

Pre- and post-test data will be collected from all groups to evaluate the effectiveness of the interventions and to evaluate the data over time. This will help ensure accurate quantitative measurement and enable better-informed conclusions and recommendations for the success and sustainability of the intervention (Briñol et al., 2020).

#### 2.8.1 Outcome measures

Evaluating the effectiveness of the intervention (i.e., proximal outcomes) will help to determine its success and whether the desired results have been attained. Proximal benefits refer to shorter-term, more immediate, subjective benefits experienced or observed, often as a direct cause of the intervention (i.e., differences in need crafting behavior and perceived managerial support). By contrast, distal outcomes are usually only noticeable over a more extended period of time and often more indirectly as a result of proximal benefits (i.e., changes in need satisfaction).

On the day of training, but before the need crafting workshop starts, participants will need to complete an outcome measure to determine their degree of need crafting, current need satisfaction, and perceived need-support from their immediate managers. The three measuring instruments to be used for the effect evaluation are discussed below (De Angelis et al., 2020). These instruments were selected for their direct alignment with the constructs in the intervention’s program theory and their operationalization, as well as for their explicit grounding in SDT. The adapted NCS operationalizes the proximal outcome targeted by NC (need crafting) and has been validated in the South African work context (Putter et al., 2024). The TMIB-S operationalizes the proximal outcome targeted by NN (perceived need-supportive managerial behaviors) and was developed and validated from an SDT perspective (Huyghebaert-Zouaghi et al., 2023). The single-item need satisfaction scales capture the distal outcome (basic psychological need satisfaction) while minimizing respondent burden across repeated measurement waves, with validity and reliability comparable to multi-item scales (Martela and Ryan, 2026).

The first instrument will be an adapted version of the Need Crafting Scale (NCS) (Laporte et al., 2021), comprising nine items. The original scale was adapted by Putter et al. (2024) to better suit the work context by making small changes to the instructions as well as the wording of items (i.e., references to “in their lives” were changed to “at work,” and “people” were replaced with “colleagues”). Only the three action-oriented items of each subscale will be administered, while the negatively phrased items will be omitted. Previous research found no significant value added from the negatively phrased items, as these items did not load significantly onto their *a priori* factors when controlling for the method factor (Putter et al., 2024). These nine items will be rated on a five-point Likert scale, ranging from 1 (completely not true/disagree) to 5 (completely true/agree). Examples of the items include “*I deliberately choose activities I am good at*” (competence crafting action), “*As much as possible, I try to do things that I really want to do rather than things that have to be done*” (autonomy crafting action), and “*Even when I feel lonely, I still try to contact colleagues who care for me*” (relatedness crafting action).

The second instrument will be the Tripartite Measure of Interpersonal Behaviors-Supervisor (TMIB-S), comprising 22 items, which will measure need-oriented leader behaviors (Huyghebaert-Zouaghi et al., 2023). However, only the “need-supportive behaviors” subscale that comprises eight items will be used to determine the degree to which managers are perceived as supportive following the intervention. Examples of items include “*My supervisor shows that they understand my perspective*” (autonomy support), “*My supervisor recognizes my efforts and accomplishments*” (competence support), and “*My supervisor shows care and concern*” (relatedness support). The need-supportive behaviors subscale will be answered through a seven-point Likert scale, ranging from 1 (strongly disagree) to 7 (strongly agree) (Huyghebaert-Zouaghi et al., 2023). These three instruments will be administered at T2 to T4 (Groups 1 and 2) and T4 to T6 (Group 3).

The last instrument will be the Single-Item Scales for Basic Psychological Need Satisfaction, comprising three items (Martela and Ryan, 2026). These items are “*I am able to do things that I really want and value at work*” (autonomy), “*I can do things well and achieve my goals*” (competence), and “*I feel close and connected with other colleagues who are important to me*” (relatedness). This three-item scale was found to be valid and reliable with strong comparability to multi-item scales. Items will be measured on a seven-point Likert scale, ranging from 1 (very strongly disagree) to 7 (very strongly agree) (Martela and Ryan, 2026).

These same three instruments, as discussed above, will be administered during the 2-weeks reflection meeting held with participants as well as 1 month after the intervention training. These outcome measures will form part of the post-test and will be administered separately to all three groups at different times (first to Group 1 and 2 and then to Group 3) in order to determine the potential changes within each group as well as measure any possible differences between the groups. Each group will undergo two separate post-tests, with all post-testing occurring between T3 and T6 (see Figures 1, 2).

#### 2.8.2 Effect evaluation data analysis plan

Background characteristics will be presented by means of descriptive statistics. Demographic characteristics (i.e., participants’ educational level, age, gender, ethnicity, organization and job tenure, duration reporting to manager, and department) will be used to determine the representativeness of the study sample and potentially to control for their impact on the effects (Bernerth and Aguinis, 2016).

We articulated the following directional hypotheses (H1 to H3) to evaluate the effectiveness of the intervention:

Hypothesis 1a (within-group): both Group 1 (dual-level) and Group 2 (employee-only) will show significant increases in need crafting behaviors from baseline to the 2-weeks and 1-month post-intervention follow-ups.

Hypothesis 1b (between-group): Group 1 will show significantly greater increases in need crafting behaviors compared to Group 2 at the 2-weeks and 1-month follow-ups.

Hypothesis 1c (sustainability): Group 1 will maintain higher levels of need crafting from the 2-weeks to the 1-month follow-up, while Group 2 may show some decline.

Hypothesis 2a (within-group): Group 1 will show significant increases in perceived need-supportive leadership behaviors from baseline to the 2-weeks and 1-month follow-ups, while Group 2 will show minimal or no change.

Hypothesis 2b (between-group): Group 1 will show significantly higher perceived need-supportive leadership behaviors compared to Group 2 at the 2-weeks and 1-month follow-ups.

Hypothesis 2c (sustainability): Group 1 will maintain elevated levels of perceived need-supportive leadership from the 2-weeks to the 1-month follow-up, while Group 2 will remain stable at baseline levels.

Hypothesis 3a (within-group): both groups will show increases in need satisfaction from baseline to the 2-weeks and 1-month follow-ups.

Hypothesis 3b (between-group): Group 1 will show significantly greater improvements in need satisfaction compared to Group 2 at the 2-weeks and 1-month follow-ups.

Hypothesis 3c (sustainability): Group 1 will maintain or further improve need satisfaction from the 2-weeks to the 1-month follow-up, while Group 2 effects may diminish over time.

Based on theoretical proximity to training content and previous research, the largest between-group differences were expected for perceived need-supportive leadership (the direct target of the leader intervention), followed by need satisfaction (downstream of both crafting and leader support), with more modest effects anticipated for need crafting behaviors (since both groups directly received crafting training).

In the intervention study, linear mixed-effects models (LMMs) will be used to evaluate the effects of interventions on need crafting behaviors, need satisfaction, and need-supportive leader behaviors (i.e., to test these hypotheses). LMMs are ideal for this design because they handle repeated measures (pre-test, post-test, and follow-up) and account for the nesting of participants within departments, addressing both individual and group-level variability. By modeling random effects for participants and departments, LMMs ensure that differences in responses over time and between groups are properly accounted for. Additionally, LMMs effectively manage missing data, allowing us to test interaction effects between time and group and providing a robust evaluation of intervention outcomes across multiple levels (e.g., Snijders and Bosker, 2012).

### 2.9 Process evaluation

It is important to note that the success of the intervention is not only dependent on its inherent qualities or characteristics. Instead, the success and subsequent outcomes of the intervention are also influenced by contextual factors (e.g., work environment and participant engagement) (Briñol et al., 2020). Consequently, process evaluation will be conducted throughout the study to determine contextual factors and their possible impact on the reach, effectiveness, adoption, implementation, and maintenance of the program of the intervention (De Angelis et al., 2020; Glasgow et al., 1999). Critical realism contends that the success of any intervention is more complex and dependent on the intricate interactions between causal mechanisms, which are different for the context of every intervention (Abildgaard et al., 2016). Critical realism does not exclude the value-add of the intervention itself. It states that these interactive mechanisms are indeed apparent in the intervention itself, but that the social, group, and organizational contexts in which the intervention is executed should not be disregarded (Spacey et al., 2019). Participants in an intervention may respond differently to the mechanisms and contexts to which they are exposed, leading to equally diverse interpretations of these. Considering the impact of factors that can constrain or enable the need-crafting intervention is vital, since the study will take place in an “open system.” Tied to the IGLO model, it will, thus, be essential to determine what factors may influence whom, why certain identified factors may have an influence, and what the potential impact of these may be on the outcomes of the study (De Angelis et al., 2020; Spacey et al., 2019). As a result, the effectiveness of the intervention will not be based solely on the content of the intervention itself, but also on the different experiences, perceptions, and interpretations of those who will participate in the intervention as well as the reasons they may respond as such (De Angelis et al., 2020; Roodbari et al., 2023).

As process evaluation will occur continuously throughout the entirety of the intervention study, fidelity measurement will be conducted through the process evaluation to determine the potential impact that various factors have on the study outcomes. Such factors include the delivery of the intervention, the degree of implementation of need crafting activities by participants, whether participants have been allowed to participate in need crafting, how useful participants have found need crafting, and whether they have, in fact, engaged with need crafting once back at their jobs (Randall et al., 2009; Spacey et al., 2019).

#### 2.9.1 Process evaluation measures

##### 2.9.1.1 Before the intervention

As part of the onboarding process – to prepare employee and managerial participants for the intervention and determine their readiness for such a study – the first process measurement will be administered. This process measurement will capture elements central to participants’ buy-in (i.e., participants’ readiness for change and communication). These and subsequent process measures will be drawn from the Intervention and Multiphase Training Transfer Assessment Questionnaire (IMTTAQ; Vasquez et al., 2026), a validated instrument that assesses contextual factors, transfer mechanisms, and behavioral outcomes across the pre-training, during-training, and post-training integration phases of occupational health training interventions. Examples of the items are “*I am ready to accept the changes brought about by the intervention*” [readiness for change; four items, adapted from Randall et al. (2009)] and “*I have received information about the goals of the intervention*” (communication; three items). These will be measured on a five-point Likert scale, ranging from 1 (strongly disagree) to 5 (strongly agree).

It will, furthermore, also be important to determine participants’ degree of job resources (i.e., autonomy) and job demands (i.e., workload). Example items are “*I am given a lot of freedom to decide how to do my own work*” and “*I never seem to have enough time to get everything done in my job*” [NIOSH Worker Well-Being Questionnaire (WellBQ)] (Chari et al., 2024). These two items will be measured on a four-point Likert scale, ranging from 1 (strongly disagree) to 4 (strongly agree). These two, as well as the above seven items, will be collected at T1 (Groups 1, 2, and 3) and again at T3 (Group 3) (see Figure 1).

##### 2.9.1.2 During the intervention

On the day of the workshop, a survey to determine the level of voluntary participation (single item), integration (three items; participants’ willingness to use what they have learnt; i.e., intention to transfer), training design (four items; transferability to the job), acceptability of the content (three items; effectiveness of materials and methods in facilitating participant learning), and the need supportiveness of the facilitator (eight items) will be administered. Participants will be asked whether they were allowed to participate voluntarily in the study, with responses to “*My participation in this intervention is voluntary*” ranging from 1 (strongly disagree) to 5 (strongly agree). Other examples include “*I believe what I learned in the training can help me at work*” (integration), “*It is clear to me that the trainer conducting the training understood how I will use what I learn*” (training design), and “*I feel the training met my expectations*” (acceptability of content) (Vasquez et al., 2026). This will be measured at T2 (for Groups 1 and 2) and T4 (for Group 3), directly after participants have received the intervention training. The trainer’s need-supportiveness will be measured through an adapted version of the Tripartite Measure of Interpersonal Behaviors-Supervisor (TMIB-S) (Huyghebaert-Zouaghi et al., 2023). Examples of items include “*The facilitator showed that he understands my perspective*” (autonomy support), “*The facilitator recognized my efforts and accomplishments*” (competence support), and “*The facilitator showed care and concern*” (relatedness support). Participants will rate these behaviors on a seven-point Likert scale, ranging from 1 (strongly disagree) to 7 (strongly agree).

##### 2.9.1.3 Implementation

After participants have had 2 weeks to implement their need crafting and need-support plans, a follow-up meeting will be arranged to measure participants’ opportunities to integrate (three items; tasks/situations needed for application), training transfer (three items; the frequency with which they apply new skills), and peer support (three items; support in applying the newly acquired skills and knowledge). Measured at T3 and T5, respectively (see Figures 1, 2), the survey will contain nine items and will be rated on a five-point Likert scale, ranging from 1 (strongly disagree) to 5 (strongly agree). Examples include “*I had access to the necessary resources to implement my need crafting plan*” (opportunities to integrate), “*I successfully applied the need crafting strategies from training in my daily work*,” and “*My colleagues asked if I had problems or trouble at work*” (Vasquez et al., 2026). Lastly, participants will be asked to share their experiences (through open-ended questions in a focus group setting) regarding the effectiveness of the implementation process and how they can ensure that the need crafting actions are sustainable and provide prolonged implementation and learning (i.e., “*What made it difficult to implement your need crafting plan?*”, “*What did you learn?*”, “*What would you regard as a developmental area for yourself?*”, and “*What was surprising for you?*”).

##### 2.9.1.4 Follow-up

Lastly, 1 month after the intervention training, participants will be required to reflect on the month that followed the intervention training and complete the same nine-item process measure as at the time of the 2-weeks reflection meeting. However, this time, an electronic version of the open-ended survey will be distributed to participants and measured at T4 and T6, respectively (see Figures 1, 2).

The IPEF will help ensure a cost-effective process evaluation through its expansive approach. The quantitative process measures also align with the validated temporal structure of the IMTTAQ (pre-training, during training, and training integration), supporting comparability with other multilevel occupational health interventions (Vasquez et al., 2026). By means of this approach, quantitative process evaluation will be done through the continuous collection of information throughout the study and follow-up questionnaires after the study (Nielsen et al., 2023). Quantitative evaluation can also be enhanced through qualitative semi-structured interviews to gather the perceptions and experiences of participants about the intervention (Abildgaard et al., 2016). The current study will primarily apply quantitative process evaluation, as it allows for standardized measures to be used. Such measures reduce the time for data collection. Quantitative process evaluation is equipped for statistical analysis and explanation of variances in outcomes, offers more breadth and representativeness of the population, and can be done at multiple intervention levels (Abildgaard et al., 2016; Randall et al., 2009). Quantitative data will be collected at both the individual and leader levels of the IGLO. However, qualitative data collection methods may be deployed for evaluation purposes in instances where discrete contextual or unexpected events may have occurred during the intervention (Nielsen et al., 2023).

To integrate process evaluation and outcome evaluation, Nielsen et al. (2023) advise that the different elements of the intervention be measured more than once during the intervention and, subsequently, analyzed to determine the influence these elements have on each other.

### 2.10 Proposed integration of effect and process evaluation

To ensure a comprehensive evaluation, the planned study will integrate effect evaluation with a realist-informed process evaluation. The evaluation of the effect will focus on whether the intervention yields the anticipated improvements in need crafting, perceived leader support, and need satisfaction, as well as whether these changes differ between groups over time. Complementing this, the realist evaluation will be used to explore how and why such outcomes may emerge, for whom they are most salient, and under what contextual conditions they are likely to occur.

#### 2.10.1 Context, mechanism, and outcome theoretical framework

From a critical realist evaluation perspective, it is not enough to state whether an intervention was successful; it is also necessary to understand why it worked or did not work, for whom, and under what circumstances (Abildgaard et al., 2016; Nielsen and Abildgaard, 2013; Roodbari et al., 2023). Such testing and evaluation of organizational interventions and their specific working mechanisms in certain contexts to yield specific outcomes are done by developing and testing context-mechanism-outcome (CMO) configurations (Nielsen and Abildgaard, 2013; Roodbari et al., 2023). The first of the three elements comprising the CMO is context: the context and environment in which the intervention occurs, which can include employees’ perceptions about the intervention, the organizational setting and culture, or available resources; these all influence how the intervention works. The second element is mechanism: the processes that are triggered and activated because of the intervention and how participants, ultimately, interpret and respond to the intervention, which drives the change. The third element is outcome, which refers to the outcomes – both anticipated and unexpected – that arise when certain mechanisms are activated in certain contexts (Roodbari et al., 2023).

#### 2.10.2 Proposed context-mechanism-outcome configurations

Guiding the protocol are preliminary program theories articulated as CMO propositions, which set out the circumstances under which intervention mechanisms are expected to be activated and produce outcomes. Context will be conceptualized as omnibus (relatively stable, broad organizational features such as workload, autonomy, or managerial participation) and discrete (situational, time-bound, and concurrent events such as fluctuating colleague support). Mechanisms will be framed as participants’ interpretations and behavioral responses (e.g., perceived relevance, opportunities for transfer, or sense of support). At the same time, outcomes will be distinguished between proximal indicators (need crafting behaviors and perceived leader support) and distal indicators (basic psychological need satisfaction).

Table 3 presents the configuration of potential CMO families that will be explored in the intervention study. The table organizes the measured variables (from Figure 1) into five higher-order families to provide a holistic framework for understanding how the intervention is expected to function. For each family, relevant contexts (e.g., readiness for change, job resources, colleague support, and trainer behaviors) are specified as the conditions within which the intervention unfolds. These contextual features are expected to trigger specific mechanisms, captured through participants’ responses such as voluntary participation, training transfer, engagement with need crafting, or perceived intervention effectiveness. When these mechanisms are activated in particular contexts, they are hypothesized to generate proximal outcomes (i.e., need crafting behaviors and perceived leader support), which, in turn, contribute to the distal outcome of basic psychological need satisfaction. This structuring highlights how different aspects of the organizational setting and intervention delivery may interact with participants’ interpretations to produce both immediate and longer-term effects.

**TABLE 3:** Context-mechanism-outcome (CMO) families for the intervention study.

CMO family	Contexts	Mechanisms	Outcomes
1. Organizational readiness and communication	- Readiness for change - Communication	- Voluntary participation - Integration (willingness to use learning)	Proximal: perceived leader support Distal: need satisfaction
2. Work environment (resources and demands)	- Job resources (autonomy) - Job demands (workload)	- Training transfer (employee)	Proximal: need crafting behaviors Distal: need satisfaction
3. Social support dynamics	- Colleague support	- Training transfer (employee)	Proximal: perceived leader support and sustained need-supportive behaviors Distal: need satisfaction
4. Training design and delivery	- Training design - Acceptability	- Integration (willingness) - Training transfer (employee and manager, as applicable)	Proximal: need crafting behaviors Distal: need satisfaction
5. Trainer facilitation	- Trainer need-supportive behaviors	- Voluntary participation - Integration (willingness)	Proximal: need crafting behaviors Distal: need satisfaction

These program theories will guide the subsequent statistical analyses by offering testable propositions about both the impact of the intervention and the contextual factors shaping its effectiveness.

#### 2.10.3 Statistical integration plan

Data will be analyzed to examine how contextual conditions interact with mechanisms to generate proximal and distal outcomes. Descriptive statistics will, firstly, be used to summarize baseline characteristics of participants and study variables. Reliability analyses will be conducted for all multi-item scales. Missing data will be handled using full information maximum likelihood (FIML), and sensitivity analyses will be conducted to assess the robustness of findings. Where appropriate, effect sizes and confidence intervals will be reported alongside statistical significance.

For CMO analyses, moderation models will be estimated to test whether contextual variables (e.g., readiness for change, communication, autonomy, workload, colleague support, training design, and trainer behaviors) influence the strength of relationships between the intervention and mechanisms (e.g., voluntary participation, training transfer, and engagement with need crafting). In turn, mediation models will be used to examine whether mechanisms predict proximal outcomes (need crafting behaviors and perceived leader support), which then lead to the distal outcome (basic psychological need satisfaction).

The qualitative data collected from the focus groups and online surveys will undergo conventional content analysis where textual data will be used to inductively develop categories and themes that best describe the data (Hsieh and Shannon, 2005). After keywords and themes have been identified, interpretation of the context will occur (Abildgaard et al., 2016). Qualitative themes will be linked to the five CMO families to confirm or expand mechanisms.

## 3 Discussion

This study presented a detailed protocol for a quasi-experimental mixed-methods intervention designed to enhance psychological need satisfaction in a South African mining organization. To our knowledge, this is the first protocol for a need-crafting intervention study designed specifically for the organizational context, uniquely combining a top-down approach (NN) with a complementary bottom-up approach (NC) to optimize need-based experiences. Aligned with positive psychology, the protocol takes a capability-building, health-promotion stance that seeks to increase everyday opportunities for autonomy, competence, and relatedness rather than remediate deficits (Sheldon and Ryan, 2011; Van der Vaart and Van den Broeck, 2019).

The implementation of previous need-crafting studies was primarily at the individual level (see Behzadnia and FatahModares, 2020, 2023; Laporte et al., 2024; van den Bogaard et al., 2024; Weinstein et al., 2016) and typically outside of the work environment. Given that work settings present unique contextual influences (Mokgata et al., 2023; Putter et al., 2024), our protocol explicitly examines need crafting under traditional employment circumstances rather than exceptional stress conditions (e.g., COVID-19 or refugee contexts) (see Behzadnia and FatahModares, 2020; Weinstein et al., 2016). Furthermore, by including a standardized intervention protocol (Slemp et al., 2021) and capturing multilevel data from both managers and employees (De Lange et al., 2024), we aim to address previous mixed findings and contribute to clearer conclusions regarding the effectiveness and contextual sensitivity of need-crafting interventions. By focusing on both proximal outcomes – immediate changes in need-crafting behaviors, and perceived managerial need-support – and distal outcomes – longer-term improvements in need satisfaction, well-being, and retention – our dual evaluation strategy (process and outcome evaluations) (Nytrø et al., 2000) provides an opportunity to explore not only whether the intervention works, but for whom, how, and under what organizational circumstances its effectiveness is maximized (Nielsen and Miraglia, 2016). In positive psychology terms, NN targets the work context as the unit of change, aiming to increase the density of need-supportive moments that enable flourishing over time.

Given ethical considerations around withholding intervention opportunities, a delayed experimental group replaces a traditional control group (Putter et al., 2024). We acknowledge potential challenges associated with delayed access, such as reduced motivation and higher dropout (Laporte et al., 2021). Additionally, with increased assurance of participant anonymity, the study seeks to minimize social desirability bias; however, complete avoidance is unlikely in social-science research where subjective interpretations of contexts occur from a personal perspective (Podsakoff et al., 2024).

Considering managers’ significant role in employee well-being – and common managerial uncertainty around providing well-being support – this intervention also deliberately aims to equip managers with practical skills to facilitate employees’ need crafting. If effective, the program can equip organizations with structured tools to proactively enhance employee engagement, well-being, and retention, treating these as downstream outcomes of improved need-supportive design and leadership (Baard et al., 2004; Gagné and Deci, 2005; Slemp et al., 2021). From a theoretical standpoint, the research may refine and extend SDT by demonstrating the complementary roles of managerial support (NN) and employee-driven need crafting (NC), consistent with SDT’s multilevel view of how social contexts shape internalization and high-quality motivation (Ryan and Deci, 2017; Vansteenkiste et al., 2020).

Ultimately, this protocol seeks to bridge theoretical frameworks with real-world application, providing an initial yet systematic exploration of the organizational conditions that enable effective need-crafting and need-support interventions. By documenting mechanisms and context conditions in a non-WEIRD workplace, and by specifying SDT-consistent pathways to be tested in subsequent analyses, the study contributes to an inclusive, practically useful evidence base for Positive Psychological Interventions at work (Duan et al., 2022; Nielsen and Miraglia, 2016).

### 3.1 Limitations

Several limitations of the proposed protocol should be emphasized. First, the design is quasi-experimental: although departments will be randomized to the immediate (Group 1) and delayed (Group 3) conditions, Group 2 will comprise departments whose managers do not volunteer and are therefore self-selected. Pre-existing differences between Group 2 and the randomized groups (e.g., in leadership quality or departmental climate) may confound between-group comparisons, as manager willingness to participate may in itself signal a more need-supportive environment. Second, the study will be conducted at a single site of one South African mining services organization and is restricted to white-collar employees, which constrains the generalizability of findings to blue-collar workers – for whom safety protocols structurally limit crafting opportunities – and to other industries, labor markets, and cultural contexts. Third, although the *a priori* power analysis indicates that 26–51 participants per group are required to detect large-to-medium effects, this calculation assumes independent observations; because participants are clustered within departments, non-trivial intraclass correlations will reduce the effective sample size and statistical power, and attrition across the six measurement waves may compound this. Because the planned linear mixed-effects models include department-level random effects, clustering is accounted for analytically rather than biasing estimates; the residual concern is statistical power, and the study may only be adequately powered to detect moderate-to-large effects. Fourth, because all groups will be drawn from a single organization, contamination is possible: employees and managers from different conditions interact daily and may share intervention content, diluting between-group differences.

Fifth, all outcome and process measures are self-reported, raising concerns about common method bias and socially desirable responding (Podsakoff et al., 2024); temporal separation of measurements and guaranteed anonymity should attenuate, but cannot eliminate, these risks. Self-report nonetheless remains the theoretically appropriate source for the focal constructs: need satisfaction is an internal experience, and need-supportive leadership is theorized to operate through employees’ perceptions of support. Objective indicators (e.g., absenteeism or turnover records) lie further downstream and depend on organizational record release and are therefore reserved for future research. Sixth, the follow-up period extends only to 1 month after training, which limits conclusions about the sustainability of intervention effects. This horizon reflects the terms of the research agreement with the host organization and the need to limit attrition and organizational-change confounds across an already intensive six-wave measurement design; proximal behavioral outcomes are expected to change within weeks, whereas longer-term sustainability is explicitly positioned as a direction for future research. Seventh, although validated and pragmatic for repeated administration, the single-item need satisfaction measures (Martela and Ryan, 2026) limit the modeling of measurement error and of subdimensions of need satisfaction. Finally, the intervention targets only the individual and leader levels of the IGLO framework (Nielsen et al., 2017); group- and organizational-level factors are measured as context but not intervened upon, and the facilitator’s dual role as researcher may introduce demand characteristics.

### 3.2 Directions for future research

The protocol also suggests several directions for future research. First, researchers should replicate the intervention in other sectors, occupational groups, and cultural contexts – including blue-collar and safety-critical roles excluded here – to establish the boundary conditions of need crafting where task discretion is structurally constrained, and to compare WEIRD and non-WEIRD settings (Duan et al., 2022). Second, follow-up periods should be extended to 6 or 12 months to evaluate whether proximal gains in need crafting and need-supportive leadership are maintained and whether distal effects on need satisfaction consolidate or decay. Third, subsequent studies should extend the intervention to the group and organizational levels of the IGLO framework (Nielsen et al., 2017) – for example, through team-based crafting components or the alignment of human resource policies with need-supportive principles – to test cumulative multilevel effects. Fourth, future studies should complement self-reports with objective and other-rated indicators (e.g., turnover records, absenteeism, and supervisor-rated performance) and adopt dyadic manager–employee designs that link individual managers’ participation in NN to their own subordinates’ outcomes. Fifth, the nomological network of outcomes could be broadened: work engagement, thriving at work (Arici Ozcan et al., 2023), and employees’ communicative behaviors, such as constructive voice and defensive silence (Şahin et al., 2021), are theoretically proximate consequences of need satisfaction that would refine tests of SDT’s motivational pathways. Because collegial solidarity appears to influence performance through individual psychological states such as engagement and thriving (Karataş and Çankır, 2023), future interventions might also explicitly target peer-to-peer need support as a complement to managerial support. Sixth, experience-sampling and diary designs could capture the day-to-day dynamics through which crafting attempts translate into need satisfaction, and realist interviews could refine the context-mechanism-outcome configurations proposed here. Seventh, researchers should examine moderating contextual factors – including workload, power distance (Şahin et al., 2021), and readiness for change – as well as the cost-effectiveness and scalability of digital or blended delivery formats. Finally, formal tests of intervention fidelity and dose-response relationships, using validated process measures such as the IMTTAQ (Vasquez et al., 2026), would strengthen causal attribution in future need-based intervention research.
